# Systemic therapy timing and use in patients with advanced melanoma at the end of life: A retrospective cohort study

**DOI:** 10.1111/1346-8138.17061

**Published:** 2023-12-11

**Authors:** P. E. A. van Lith, K. Schreuder, M. Jalving, A. K. L. Reyners, L. B. Been, E. Rácz, H. P. Fransen, M. W. J. Louwman

**Affiliations:** ^1^ Department of Research and Development Netherlands Comprehensive Cancer Organization Utrecht The Netherlands; ^2^ Department of Surgery University Medical Centre Groningen, University of Groningen Groningen The Netherlands; ^3^ Department of Medical Oncology University Medical Centre Groningen, University of Groningen Groningen The Netherlands; ^4^ Department of Surgical Oncology University Medical Centre Groningen, University of Groningen Groningen The Netherlands; ^5^ Department of Dermatology University of Medical Centre Groningen, University of Groningen Groningen The Netherlands; ^6^ Netherlands Association for Palliative Care (PZNL) Utrecht The Netherlands

**Keywords:** advanced melanoma, end of life, immune therapy, systemic therapy, targeted therapy

## Abstract

Novel systemic therapies for advanced melanoma improve survival, but carry potential serious side effects and high costs. This study aimed to assess the timing and use of systemic therapies in the months before death. Patients diagnosed with advanced melanoma (July 2017–June 2020) who died before July 2020 were selected from the Netherlands Cancer Registry. We evaluated the timing of systemic therapies within 30 days and 3 months before death, and studied patient and tumor characteristics associated with systemic therapy use between diagnosis and death. Out of 1097 patients 68% received systemic therapy. Almost 25% and 10% started a new therapy within 90 days and within 30 days before death, respectively. Female sex, elevated LDH, BRAF mutation, poor ECOG performance status (≥3), and high comorbidity index reduced the odds of receiving immune therapy. Poor performance status and high comorbidity decreased the odds for both therapies. A considerable number of patients started systemic therapy shortly before death, emphasizing the importance of considering potential benefits and drawbacks through shared decision‐making.

## 1 INTRODUCTION

Melanoma incidence is relatively high in the Netherlands and still increasing.^1^ Approximately 11% of the patients have an advanced melanoma (stage III or IV) at the time of primary diagnosis.^2^ The introduction of immune and targeted therapy in 2012 has dramatically changed treatment and survival rates have improved.^3^, ^4^, ^5^ However, these new therapies may lead to severe side effects and are costly,^6^, ^7^ underscoring the need for careful evaluation, especially towards the end of life.^6^, ^8^

This study aimed to examine the timing and utilization of systemic therapy in advanced melanoma patients shortly before death, and evaluate the patient and tumor characteristics associated with its use. Towards the end of life, this information provides valuable insights to optimize shared decision‐making.

## 2 PATIENT AND METHODS

### 2.1 Data source

Data were retrieved from the population‐based Netherlands Cancer Registry (NCR), which is hosted by the Netherlands Comprehensive Cancer Organization. Their trained data managers collect patient, tumor, diagnostic and treatment characteristics for all cancer patients in the Netherlands. Since July 2017, additional data have been collected for all (synchronous and metachronous) advanced‐stage melanoma patients.^9^

### 2.2 Study population

All advanced melanoma patients diagnosed between July 2017 and June 2020 (*N* = 3687) who died before July 1, 2020 (*N* = 1097) were included. Patients who received chemotherapy (*N* = 3) and those with uveal or mucosal melanoma (*N* = 53) were excluded. Advanced‐stage definition changed according to Dutch therapy guidelines: stage IV from July 2017, inoperable stage IIIC/IIID from July 2017, all stage IIIC/IIID from July 2018, and all stage IIIA/IIIB from January 2019. Low‐stage patients were included if they progressed to advanced melanoma in the specified period. In the Netherlands, health insurance covers systemic therapies for diagnosed advanced melanoma patients.

### 2.3 Data analysis

Characteristics at diagnosis were described: age, sex, topography and morphology (according to ICDO, 3rd edition), TNM stage (UICC, 8th edition), synchronous versus metachronous metastasis, elevated LDH levels (>250 U/L), elevated S100B levels (>0.10 μg/L; a prognostic biomarker for melanoma relapse and mortality risk), BRAF mutation, ECOG performance status, Charlson comorbidity index, number of distant metastases, diagnosed in a melanoma center, type of systemic therapy, the interval between primary and advanced melanoma diagnosis (categorized by approximately equal group size ≤36 or >36 months), and survival after advanced melanoma diagnosis.

We stratified according to whether or not patients received systemic therapy, and some analyses were performed according to period before death (within 30 or 31–90 days).

Logistic regression assessed patient and tumor characteristics related to systemic therapy use between advanced melanoma diagnosis and death. Analyses on receiving targeted therapy and both therapies were limited to patients with a BRAF mutation. Chi‐square and rank‐sum tests were performed for categorical variables and median values, respectively. *P* values <0.05 were considered statistically significant. Analyses were performed using STATA (StataCorp. 2019, Stata Statistical Software Release 16.1; College Station, TX, USA).

## 3 RESULTS

In total, 744 patients out of 1097 deceased advanced melanoma patients (67.8%) were treated with systemic therapy. Systemically treated patients were younger (68 vs. 76 years), more often had normal LDH levels (40.5% vs. 24.7%), BRAF mutation was tested more often (*P* < 0.001), performance status was better (*P* < 0.001), and comorbidity was more prevalent (*P* < 0.001; Table 1). Median survival time was significantly longer for systemically treated patients (8.4 vs. 2.3 months).

**TABLE 1:** Patient and tumor characteristics at diagnosis of patients diagnosed with advanced melanoma during 2017–2020 and deceased before July 1, 2020.

	All patients (*N* = 1097)	Systemic treatment (*N* = 744)	No systemic treatment (*N* = 353)	*p*‐Value**
Age, years (median, range)	71 (24–97)	68 (24–93)	76 (39–97)	0.000*
Age category				
≤40	37 (3.4%)	36 (4.9%)	1 (0.3%)	0.000*
41–54	129 (11.8%)	111 (14.9%)	18 (5.1%)	
55–64	187 (17.0%)	149 (20.0%)	38 (10.8%)	
65–74	335 (30.5%)	248 (33.3%)	87 (24.6%)	
≥75	409 (37.3%)	200 (26.9%)	209 (59.2%)	
Sex				
Male	698 (63.6%)	485 (65.2%)	213 (60.3%)	0.12
Female	399 (36.4%)	259 (34.8%)	140 (39.7%)	
Topography				
Skin	936 (85.3%)	650 (87.4%)	286 (81.0%)	0.01*
Unknown primary site	161 (14.7%)	94 (12.6%)	67 (19.0%)	
Morphology				
Superficial spreading melanoma	513 (46.8%)	368 (49.5%)	145 (41.1%)	0.06
Nodular melanoma	249 (22.7%)	159 (21.4%)	90 (25.5%)	
Malignant melanoma NOS	271 (24.7%)	173 (23.2%)	98 (27.7%)	
Other	64 (5.8%)	44 (5.9%)	20 (5.7%)	
Synchronous or metachronous metastasis				
Synchronous	241 (22.0%)	154 (20.7%)	87 (24.7%)	0.13
Metachronous	817 (74.5%)	567 (76.2%)	250 (70.8%)	
Unknown	39 (3.5%)	23 (3.1%)	16 (4.5%)	
Elevated LDH serum at diagnosis (>250 U/L)				
Yes	548 (50.0%)	366 (49.2%)	182 (51.5%)	0.000*
No	388 (35.3%)	301 (40.5%)	87 (24.7%)	
Unknown	161 (14.7%)	77 (10.3%)	84 (23.8%)	
Elevated serum S100B (>0.10 μg/L)				
Yes	252 (23.0%)	219 (29.4%)	33 (9.3%)	0.000*
No	118 (10.8%)	91 (12.2%)	27 (7.7%)	
Unknown	727 (66.2%)	434 (58.4%)	293 (83.0%)	
BRAF mutation				
Negative	429 (39.1%)	290 (39.0%)	139 (39.4%)	0.000*
Positive	450 (41.0%)	383 (51.5%)	67 (19.0%)	
Unknown/not tested	218 (19.9%)	71 (9.5%)	147 (41.6%)	
ECOG performance status				
0	300 (27.4%)	271 (36.4%)	29 (8.2%)	0.000*
1	311 (28.4%)	250 (33.6%)	61 (17.3%)	
2	127 (11.5%)	85 (11.4%)	42 (11.9%)	
3 or above	87 (7.9%)	31 (4.2%)	56 (15.9%)	
Unknown	272 (24.8%)	107 (14.4%)	165 (46.7%)	
Charlson comorbidity index				
0	447 (40.7%)	341 (45.8%)	106 (30.0%)	0.000*
1	246 (22.4%)	164 (22.0%)	82 (23.2%)	
2 or more	163 (14.9%)	84 (11.3%)	79 (22.4%)	
Unknown	241 (22.0%)	155 (20.9%)	86 (24.4%)	
Number of metastases				
None/unknown	194 (17.7%)	116 (15.6%)	78 (22.1%)	0.000*
1	206 (18.8%)	117 (15.7%)	89 (25.2%)	
2	168 (15.3%)	111 (14.9%)	57 (16.1%)	
3	179 (16.3%)	132 (17.7%)	47 (13.3%)	
4	164 (15.0%)	126 (17.0%)	38 (10.8%)	
5 or more	186 (16.9%)	142 (19.1%)	44 (12.5%)	
Diagnosed in melanoma center				
Yes	744 (67.8%)	577 (77.6%)	187 (53.0%)	0.000*
No	353 (32.2%)	167 (22.4%)	166 (47.0%)	
Systemic therapy				
None	353 (32.2%)	0 (0.0%)	353 (100%)	0.000*
Immune therapy	346 (31.5%)	346 (46.5%)	0 (0.0%)	
Targeted therapy	150 (13.7%)	150 (20.2%)	0 (0.0%)	
Both	248 (22.6%)	248 (33.3%)	0 (0.0%)	
Interval between primary and advanced diagnosis				
Advanced at primary diagnosis	301 (27.4%)	184 (24.7%)	117 (33.1%)	0.001*
≤36 months	422 (38.5%)	282 (37.9%)	140 (39.7%)	
>36 months	374 (34.1%)	278 (37.4%)	96 (27.2%)	
Survival (months) after diagnosis of advanced melanoma				
Median	6.5	8.4	2.3	0.000*
Range	0–77	0.3–77	0–33	

Abbrevations: BRAF, proto‐oncogene B‐Raf; ECOG, eastern cooperative oncology group; ICDO, International Classification of Diseases for Oncology; LDH, lactate dehydrogenase; NOS, not otherwise specified; S100B, S100 calcium‐binding protein B; TNM, classification of malignant tumors; UICC, Union for International Cancer Control.

* *P* value statistically significant.

** *P* value for comparison systemic treated versus no systemic treated patients.

### 3.1 Timing of systemic therapy

In total, 263 (24.0%) of the deceased patients started a new (first) line of systemic therapy within 90 days before death. Most of them (*N* = 174) started between 90 and 31 days, and 89 (8.1%) started within 30 days prior to death (Table 2). Most received immune therapy (75.3% and 68.5%), while 23.0% and 31.5% received targeted therapy (Table 2). The timing, use, and sequence of systemic therapies are shown in Supporting Information Table S1.

**TABLE 2:** Patient and tumor characteristics at start of systemic therapy among patients with advanced melanoma during the last 90–31 days or last 30 days before death.

Start of therapy (days before death)	90–31, *N* (%)	30–0, *N* (%)
None	923 (84%)	1008 (92%)
Started with new therapy	174 (16%)	89 (8.1%)
Immune therapy	131 (75%)	61 (69%)
Targeted therapy	40 (23%)	28 (31%)
Both therapies	3 (1.7%)	0

	Immune (*N* = 131)	Targeted (*N* = 40)	Immune (*N* = 61)	Targeted (*N* = 28)
Age, years (median, range)	68 (31–93)	65 (24–93)	63 (30–89)	71 (48–90)
Age group				
≤40	4 (3.1%)	3 (7.5%)	4 (6.5%)	0 (0.0%)
41–54	18 (13.7%)	6 (15.0%)	10 (16.4%)	2 (7.1%)
55–64	20 (15.3%)	6 (15.0%)	17 (27.9%)	7 (25.0%)
65–74	46 (35.1%)	13 (32.5%)	17 (27.9%)	7 (25.0%)
≥75	43 (32.8%)	12 (30.0%)	13 (21.3%)	12 (42.9%)
Sex				
Male	91 (69.5%)	19 (47.5%)	40 (65.6%)	21 (75.0%)
Female	40 (30.5%)	21 (52.5%)	21 (34.4%)	7 (25.0%)
Elevated LDH serum (>250 U/L)				
Yes	75 (57.3%)	30 (75.0%)	36 (59.0%)	23 (82.1%)
No	43 (32.8%)	9 (22.5%)	17 (27.9%)	0 (0.0%)
Unknown	13 (9.9%)	1 (2.5%)	8 (13.1%)	5 (17.9%)
BRAF mutation				
Negative	65 (49.6%)	3 (7.5%)	30 (49.2%)	9 (32.1%)
Positive	51 (38.9%)	37 (92.5%)	23 (37.7%)	16 (57.2%)
Unknown/not tested	15 (11.5%)	0 (0.0%)	8 (13.1%)	3 (10.7%)
ECOG performance status				
0	35 (26.7%)	9 (22.5%)	19 (31.2%)	7 (25.0%)
1	53 (40.5%)	12 (30.0%)	23 (37.7%)	5 (17.9%)
2	19 (14.5%)	8 (20.0%)	5 (8.2%)	3 (10.6%)
3 or above	5 (3.8%)	7 (17.5%)	4 (6.5%)	2 (7.1%)
Unknown	19 (14.5%)	4 (10.0%)	10 (16.4%)	11 (39.4%)
Charlson comorbidity index				
0	64 (48.9%)	21 (52.5%)	28 (45.9%)	10 (35.7%)
1	23 (17.6%)	7 (17.5%)	16 (26.2%)	5 (17.9%)
2 or more	16 (12.2%)	8 (20.0%)	5 (8.2%)	4 (14.3%)
Unknown	28 (21.3%)	4 (10.0%)	12 (19.7%)	9 (32.1%)
Number of metastases				
None/unknown	8 (6.1%)	3 (7.5%)	2 (3.3%)	3 (10.6%)
1	27 (20.6%)	3 (7.5%)	10 (16.4%)	9 (32.1%)
2	23 (17.6%)	8 (20.0%)	13 (21.3%)	1 (3.6%)
3	19 (14.5%)	6 (15.0%)	12 (19.7%)	5 (17.9%)
4	21 (16.0%)	4 (10.0%)	13 (21.3%)	5 (17.9%)
5 or more	33 (25.2%)	16 (40.0%)	11 (18.0%)	5 (17.9%)
Survival (months) between advanced diagnoses and death				
Median	3.7	3.4	2.6	2.2
Range	1.4–39.2	1.2–22.8	0.3–20.0	0.4–20.0

Abbrevations: BRAF, proto‐oncogene B‐Raf; ECOG, eastern cooperative oncology group; ICDO, International Classification of Diseases for Oncology; LDH, lactate dehydrogenase; NOS, not otherwise specified; S100B, S100 calcium‐binding protein B; TNM, classification of malignant tumors; UICC, Union for International Cancer Control.

### 3.2 Systemic therapy (immune, targeted or both)

Multivariable analyses demonstrated that patients under 40 had an odds ratio of 15.2 (95% confidence interval [CI] 1.8–129) for receiving systemic therapy compared to those aged above 75 (Table 3).

**TABLE 3:** Regression analyses for the association with systemic therapy use among patients with advanced melanoma.

	Univariable regression		*P* value	Multivariable regression		*P* value
	Odds ratio	95% CI		Odds ratio	95% CI	
Age category						
≤40	37.6	5.11–276	0.000*	15.2	1.78–129	0.01*
41–54	6.44	3.78–11.0	0.000*	3.02	1.60–5.69	0.001*
55–64	4.10	2.73–6.15	0.000*	2.32	1.41–3.82	0.001*
65–74	2.98	2.18–4.07	0.000*	1.71	1.16–2.50	0.01*
≥75	1.00	–	–	–	–	–
Sex						
Male	1.00	–	–	–	–	–
Female	0.81	0.63–1.05	0.12	–	–	–
Topography						
Skin	1.00	–	–	1.00	–	–
Unknown primary site	0.62	0.44–0.87	0.01*	0.78	0.39–1.54	0.47
Morphology						
Superficial spreading melanoma	1.00	–	–	1.00	–	–
Nodular melanoma	0.70	0.50–0.96	0.03*	0.73	0.49–1.11	0.14
Malignant melanoma NOS	0.70	0.51–0.95	0.02*	0.76	0.43–1.34	0.34
Other	0.87	0.49–1.52	0.62	1.07	0.53–2.12	0.86
Synchronous or metachronous metastasis						
Metachronous	1.00	–	–	–	–	–
Synchronous	0.78	0.58–1.06	0.11	–	–	–
Unknown	0.63	0.33–1.22	0.17	–	–	–
Elevated serum LDH level (>250 U/L)						
Yes	0.58	0.43–0.78	0.000*	0.71	0.48–1.05	0.09
No	1.00	–	–	1.00	–	–
Unknown	0.26	0.18–0.39	0.000*	1.43	0.79–2.61	0.24
Elevated serum S100B (>0.10 μg/L)						
Yes	1.00	–	–	1.00	–	–
No	0.51	0.29–0.89	0.02*	0.50	0.25–0.98	0.04*
Unknown	0.22	0.15–0.33	0.000*	0.38	0.23–0.61	0.000*
BRAF mutation						
Negative	1.00	–	–	1.00	–	–
Positive	2.74	1.97–3.81	0.000*	2.59	1.74–3.85	0.000*
Unknown/not tested	0.23	0.16–0.33	0.000*	0.38	0.23–0.62	0.000*
ECOG performance status						
0	1.00	–	–	1.00	–	–
1	0.44	0.27–0.70	0.001*	0.50	0.29–0.84	0.01*
2	0.22	0.13–0.37	0.000*	0.24	0.13–0.44	0.000*
3 or above	0.06	0.03–0.11	0.000*	0.05	0.03–0.11	0.000*
Unknown	0.07	0.04–0.11	0.000*	0.09	0.05–0.17	0.000*
Charlson comorbidity index						
0	1.00	–	–	1.00	–	–
1	0.62	0.44–0.88	0.01*	0.89	0.57–1.37	0.59
2 or more	0.33	0.23–0.48	0.000*	0.57	0.35–0.92	0.02*
Unknown	0.56	0.40–0.79	0.001*	1.61	0.97–2.67	0.07
Number of metastases						
None/unknown	1.00	–	–	1.00	–	–
1	0.88	0.59–1.32	0.54	0.45	0.26–0.77	0.004*
2	1.31	0.85–2.01	0.22	0.73	0.42–1.30	0.29
3	1.89	1.22–2.93	0.001*	1.06	0.59–1.90	0.85
4	2.23	1.39–3.38	0.001*	1.15	0.62–2.13	0.66
5 or more	2.17	1.39–3.38	0.001*	1.00	0.54–1.86	0.99
Interval between primary and advanced diagnosis						
Advanced at primary diagnosis	1.00	–	–	1.00	–	–
≤36 months	1.28	0.94–1.74	0.12	1.25	0.77–2.05	0.37
>36 months	1.84	1.33–2.56	0.000*	1.01	0.59–1.71	0.97

Abbrevations: BRAF, proto‐oncogene B‐Raf; ECOG, eastern cooperative oncology group; ICDO, International Classification of Diseases for Oncology; LDH, lactate dehydrogenase; NOS, not otherwise specified; S100B, S100 calcium‐binding protein B; TNM, classification of malignant tumors; UICC, Union for International Cancer Control.

* *P* value statistically significant.

For immune therapy, the odds were lower for females (odds ratio [OR] = 0.57 [0.40–0.82]), those with elevated LDH (OR = 0.64 [0.43–0.94]), BRAF‐positive patients (OR = 0.03 [0.02–0.04]), and patients with poor performance status (OR = 0.11 [0.05–0.24]) and more comorbidities (OR = 0.41 [0.24–0.71]). The odds for receiving targeted therapy were higher for female patients (OR = 1.56 [1.05–2.30]), patients with elevated S100B, and patients with poor performance status (OR = 2.31 [1.12–4.77]). The odds for receiving both immune and targeted therapy were highest for patients younger than 40 (OR = 6.30 [2.77–14.4]; Supporting Information Tables S2 and S3).

## 4 DISCUSSION

This population‐based nationwide study aimed to examine the timing and use of systemic therapies among advanced melanoma patients shortly before death. Almost 70% of patients in this real‐world cohort received systemic therapy between advanced melanoma diagnosis and death, with a considerable proportion starting shortly before death.

### 4.1 Timing

We found that 25% of patients started a new systemic therapy within 90 days prior to death, similar to a recent study (29%) that included treated patients exclusively.^10^

Surprisingly, 8.1% of patients started a new systemic therapy within 30 days prior to death. Immune therapy was possibly started shortly before death because clinicians and patients overestimated the effectiveness of prolonging life, disregarding that response to immune therapy requires time.^11^ In contrast, targeted therapy, known for its rapid response, may be used for palliative symptom relief.^12^ We found that only 28 out of 89 patients started with a first (new) line of targeted therapy within 30 days prior to death, which is less than a previous study in which most BRAF‐positive patients received targeted therapy during the last months of life.^13^ However, those were patients with brain metastases only and the study also included therapy that started prior to the last 3 months of life.

### 4.2 Immune therapy

Males received immune therapy more often than females, which is likely a reflection of gender differences in other baseline characteristics. Patients who started immune therapy had better performance status and less comorbidities. This is not surprising as immune therapy could have serious side effects,^6^ although a recent study found a similar response to immune therapy in patients with and without comorbidities.^14^

### 4.3 Targeted therapy

We found that patients with a poor performance status were more likely to receive targeted therapy. This is probably because the side effects of targeted therapy are in general less severe compared to the side effects of immune therapy. Also, targeted therapy induces faster response and therefore also serves a palliative purpose in terms of quality of life preservation.^12^, ^13^

Starting or ending systemic therapy is complicated and often emotionally charged, especially when the patient is approaching the end of life. Decisions should be made through a well‐informed assessment that considers patient and tumor characteristics, combined with the patient's and their family's preferences to optimize personalized care in the months before death.^15^

### 4.4 Strengths and limitations

This study is based on NCR data, which are uniformly and independently gathered among all cancer patients in the country. Moreover, patients with advanced melanoma whether receiving any treatment or not were included. This increases the generalizability of the results and provides a good reflection of daily practice.

Some limitations of this study should be noted. First, including the first 6 months of 2020 could lead to selection bias due to limited follow‐up time. We therefore performed our analyses excluding 2020, which showed similar results. Secondly, follow‐up data from one melanoma center lacked, but we included 13 of the 14 melanoma centers in the Netherlands. Lastly, no data on factors such as patient or family preference and quality of life were available, which likely influenced treatment choices.

In conclusion, this study showed that a considerable number of patients started systemic therapy shortly before death. It is crucial to carefully consider the potential benefits and drawbacks of initiating these novel therapies in the final stages of life, emphasizing the importance of shared decision‐making between patients and their healthcare providers.

## CONFLICT OF INTEREST STATEMENT

All authors declare no direct conflict with this work. For unrelated conflicts, M.J. has an advisory role for Bristol Myers Squibb, Merck Sharp & Dohme, Novartis, AstraZeneca, Tesaro, and Pierre Fabre.

## ETHICS STATEMENT

According to the Central Committee on Research involving Human Subjects, this type of study does not require approval from an ethics committee in the Netherlands. This study was approved by the Privacy Review Board of the Netherlands Cancer Registry.

## Supporting information


Tables S1.

## Data Availability

The data that support the findings of this study are available from Netherlands Comprehensive Cancer Organization, but restrictions apply to the availability of these data, which were used under license for the current study and so are not publicly available. Data are available from the authors upon reasonable request and with the permission of the Netherlands Comprehensive Cancer Organization.
