# Supplementary material for: Systemic therapy timing and use in patients with advanced melanoma at the end of life: A retrospective cohort study
Source: J Dermatol. 2023 Dec 11;51(4):584–91. doi: 10.1111/1346-8138.17061 (PMC11483958; doi:10.1111/1346-8138.17061)
Supplement: Supplementary file 1 — Tables S1. [file JDE-51--s001.docx]

**TABLES**

Table 1: Timing, use and order of systemic therapies days before death among patients with advanced melanoma diagnosed 2017-2020, deceased before July 1^st^ 2020.

|  | **90-31 days** |  |  | **30-0 days** |  |
| --- | --- | --- | --- | --- | --- |
|  |  |  |  |  |  |
|  | **N** | **(%)** |  | **N** | **(%)** |
| No new start systemic therapy | 923 | 84.1% | No new treatment change | 842 | 91.2% |
|  |  |  | Immune therapy | 59 | 6.4% |
|  |  |  | Targeted therapy | 22 | 2.4% |
| Immune therapy | 131 | 11.9% | No new treatment change | 125 | 95.4% |
|  |  |  | Immune therapy | 0 | 0.0% |
|  |  |  | Targeted therapy | 6 | 4.6% |
| Targeted therapy | 40 | 3.7% | No new treatment change | 38 | 95.0% |
|  |  |  | Immune therapy | 2 | 5.0% |
|  |  |  | Targeted therapy | 0 | 0.0% |
| Both therapies | 3 | 0.3% | No new treatment change | 3 | 100% |
|  |  |  | Immune therapy | 0 | 0.0% |
|  |  |  | Targeted therapy | 0 | 0.0% |

| Table 2: Patient and tumor characteristics associated with systemic therapy use (immune, targeted or both) in advanced melanoma patients (univariable logistic regression model) | | | | | | | | | | | | |
| --- | --- | --- | --- | --- | --- | --- | --- | --- | --- | --- | --- | --- |
|  | Immune therapy (N=346) | | |  | Targeted therapy† (N=130) | | |  | Both therapies† (N=221) | | |  |
|  | N (%) | Odds Ratio | 95% CI | P-value | N (%) | Odds Ratio | 95% CI | P-value | N (%) | Odds Ratio | 95% CI | P-value |
| **Age group** |  |  |  |  |  |  |  |  |  |  |  |  |
| ≤ 40 | 5 (1.5%) | 0.36 | 0.14 - 0.95 | 0.04* | 7 (5.4%) | 2.42 | 1.01 - 5.89 | 0.05* | 19 (8.6%) | 14.4 | 6.78 - 30.4 | 0.000* |
| 41-54 | 38 (11.0%) | 0.97 | 0.63 - 1.50 | 0.89 | 12 (9.2%) | 1.06 | 0.54 - 2.11 | 0.86 | 54 (24.4%) | 9.80 | 5.82 - 16.5 | 0.000* |
| 55-64 | 59 (17.0%) | 1.07 | 0.74 - 1.56 | 0.72 | 27 (20.8%) | 1.75 | 1.03 - 2.98 | 0.04* | 48 (21.7%) | 4.70 | 2.84 - 7.79 | 0.000* |
| 65-74 | 121 (35.0%) | 1.31 | 0.97 - 1.79 | 0.08 | 48 (36.9%) | 1.73 | 1.10 - 2.74 | 0.02* | 72 (32.6%) | 3.73 | 2.34 - 5.92 | 0.000* |
| ≥ 75 | 123 (35.5%) | Ref. |  |  | 36 (27.7%) | Ref. |  |  | 28 (12.7%) | Ref. |  |  |
| **Sex** |  |  |  |  |  |  |  |  |  |  |  |  |
| Male | 236 (68.2%) | Ref. |  |  | 70 (53.9%) | Ref. |  |  | 150 (67.9%) | Ref. |  |  |
| Female | 110 (31.8%) | 0.75 | 0.57 - 0.98 | 0.03* | 60 (46.1%) | 1.59 | 1.10 - 2.30 | 0.01* | 71 (32.1%) | 0.79 | 0.58 - 1.08 | 0.14 |
| **Topography** |  |  |  |  |  |  |  |  |  |  |  |  |
| Skin | 306 (88.4%) | Ref. |  |  | 114 (87.7%) | Ref. |  |  | 188 (85.1%) | Ref. |  |  |
| Unknown primary site | 40 (11.6%) | 0.68 | 0.46 – 0.99 | 0.05* | 16 (12.3%) | 0.80 | 0.46 - 1.38 | 0.42 | 33 (14.9%) | 1.03 | 0.68 - 1.55 | 0.90 |
| **Morphology** |  |  |  |  |  |  |  |  |  |  |  |  |
| Superficial Spreading Melanoma | 153 (44.2%) | Ref. |  |  | 73 (56.2%) | Ref. |  |  | 121 (54.8%) | Ref. |  |  |
| Nodular Melanoma | 83 (24.0%) | 1.18 | 0.85 - 1.63 | 0.33 | 30 (23.1%) | 0.83 | 0.52 - 1.30 | 0.41 | 37 (16.7%) | 0.57 | 0.38 - 0.85 | 0.01* |
| Malignant Melanoma NOS | 76 (22.0%) | 0.92 | 0.66 - 1.27 | 0.60 | 24 (18.4%) | 0.59 | 0.36 - 0.95 | 0.03* | 59 (26.7%) | 0.90 | 0.63 - 1.28 | 0.57 |
| Other | 34 (9.8%) | 2.67 | 1.58 - 4.51 | 0.000* | 3 (2.3%) | 0.30 | 0.09 - 0.97 | 0.04 | 4 (1.8%) | 0.22 | 0.08 - 0.61 | 0.004* |
| **Synchronous versus metachronous** |  |  |  |  |  |  |  |  |  |  |  |  |
| Metachronous | 262 (75.7%) | Ref. |  |  | 98 (75.4%) | Ref. |  |  | 170 (76.9%) | Ref. |  |  |
| Synchronous | 73 (21.1%) | 0.92 | 0.67 - 1.26 | 0.60 | 30 (23.1%) | 1.04 | 0.67 - 1.61 | 0.85 | 44 (19.9%) | 0.85 | 0.59 - 1.23 | 0.39 |
| Unknown | 11 (3.2%) | 0.83 | 0.41 - 1.70 | 0.61 | 2 (1.5%) | 0.40 | 0.09 - 1.67 | 0.21 | 7 (3.2%) | 0.83 | 0.36 - 1.92 | 0.67 |
| **Elevated serum LDH level (>250 U/L)** |  |  |  |  |  |  |  |  |  |  |  |  |
| Yes | 151 (43.6%) | 0.59 | 0.45 - 0.78 | 0.000* | 90 (69.2%) | 1.92 | 1.27 - 2.90 | 0.002* | 112 (50.7%) | 0.70 | 0.52 - 0.95 | 0.02* |
| No | 152 (43.9%) | Ref. |  |  | 36 (27.7%) | Ref. |  |  | 104 (47.1%) | Ref. |  |  |
| Unknown | 43 (12.5%) | 0.57 | 0.38 - 0.85 | 0.01* | 4 (3.1%) | 0.25 | 0.09 - 0.71 | 0.01* | 5 (2.2%) | 0.09 | 0.03 - 0.22 | 0.000* |
| **BRAF mutation** |  |  |  |  |  |  |  |  |  |  |  |  |
| Negative | 268 (77.5%) | Ref. |  |  | 0 (0.0%) | † |  |  | 0 (0.0%) | † |  |  |
| Positive | 32 (9.2%) | 0.05 | 0.03 - 0.07 | 0.000* | 130 (100%) |  |  |  | 221 (100%) |  |  |  |
| Unknown/Not tested | 46 (13.3%) | 0.16 | 0.11 - 0.23 | 0.000* | 0 (0.0%) |  |  |  | 0 (0.0%) |  |  |  |
| **ECOG performance status** |  |  |  |  |  |  |  |  |  |  |  |  |
| 0 | 126 (36.4%) | Ref. |  |  | 26 (20.0%) | Ref. |  |  | 110 (49.8%) | Ref. |  |  |
| 1 | 129 (37.3%) | 0.98 | 0.71 - 1.35 | 0.90 | 40 (30.8%) | 1.56 | 0.92 - 2.62 | 0.10 | 75 (33.9%) | 0.55 | 0.39 - 0.78 | 0.001* |
| 2 | 31 (9.0%) | 0.45 | 0.28 - 0.71 | 0.001* | 29 (22.3%) | 3.12 | 1.75 - 5.56 | 0.000* | 22 (9.9%) | 0.36 | 0.22 - 0.61 | 0.000* |
| 3 or above | 10 (2.9%) | 0.18 | 0.09 - 0.36 | 0.000* | 16 (12.3%) | 2.37 | 1.21 - 4.67 | 0.01* | 3 (1.4%) | 0.06 | 0.02 - 0.20 | 0.000* |
| Unknown | 50 (14.4%) | 0.31 | 0.21 - 0.46 | 0.000* | 19 (14.6%) | 0.79 | 0.43 - 1.46 | 0.46 | 11 (5.0%) | 0.07 | 0.04 - 0.14 | 0.000* |
| **Charlson comorbidity index** |  |  |  |  |  |  |  |  |  |  |  |  |
| 0 | 146 (42.2%) | Ref. |  |  | 59 (45.4%) | Ref. |  |  | 123 (55.6%) | Ref. |  |  |
| 1 | 90 (26.0%) | 1.19 | 0.86 - 1.65 | 0.30 | 23 (17.7%) | 0.68 | 0.41 - 1.13 | 0.14 | 45 (20.4%) | 0.59 | 0.40 - 0.87 | 0.01* |
| 2 or more | 40 (11.6%) | 0.67 | 0.45 – 0.99 | 0.05* | 26 (20.0%) | 1.25 | 0.76 - 2.06 | 0.39 | 15 (6.8%) | 0.27 | 0.15 - 0.47 | 0.000* |
| Unknown | 70 (20.2%) | 0.84 | 0.60 - 1.19 | 0.33 | 22 (16.9%) | 0.66 | 0.39 - 1.11 | 0.12 | 38 (17.2%) | 0.49 | 0.33 - 0.74 | 0.001* |

| *Appendix II (continued)* |  |  |  |  |  |  |  |  |  |  |  |  |
| --- | --- | --- | --- | --- | --- | --- | --- | --- | --- | --- | --- | --- |
| **Socioeconomic status (SES)** |  |  |  |  |  |  |  |  |  |  |  |  |
| Low | 48 (13.9%) | Ref. |  |  | 18 (13.9%) | Ref. |  |  | 33 (14.9%) | Ref. |  |  |
| Low-middle | 41 (11.8%) | 0.77 | 0.47 - 1.27 | 0.31 | 18 (13.9%) | 0.98 | 0.49 - 1.96 | 0.95 | 40 (18.1%) | 1.26 | 0.74 - 2.14 | 0.40 |
| Middle | 47 (13.6%) | 0.93 | 0.57 - 1.52 | 0.77 | 18 (13.9%) | 0.97 | 0.48 - 1.95 | 0.93 | 38 (17.2%) | 1.16 | 0.68 - 1.99 | 0.58 |
| Middle-high | 40 (11.6%) | 0.81 | 0.49 - 1.34 | 0.41 | 17 (13.0%) | 0.98 | 0.48 - 1.98 | 0.95 | 38 (17.2%) | 1.27 | 0.74 - 2.18 | 0.39 |
| High | 49 (14.1%) | 1.10 | 0.67 - 1.81 | 0.70 | 11 (8.4%) | 0.61 | 0.28 - 1.34 | 0.21 | 28 (12.7%) | 0.86 | 0.48 - 1.51 | 0.59 |
| Unknown | 121 (35.0%) | 0.88 | 0.58 - 1.32 | 0.53 | 48 (36.9%) | 0.96 | 0.54 - 1.72 | 0.90 | 44 (19.9%) | 0.42 | 0.25 - 0.69 | 0.001* |
| **Number of metastases** |  |  |  |  |  |  |  |  |  |  |  |  |
| None/Unknown | 68 (19.6%) | Ref. |  |  | 15 (11.5%) | Ref. |  |  | 22 (10.0%) | Ref. |  |  |
| 1 | 59 (17.1%) | 0.74 | 0.49 - 1.13 | 0.17 | 14 (10.8%) | 0.87 | 0.41 - 1.85 | 0.72 | 35 (15.8%) | 1.60 | 0.90 - 2.84 | 0.11 |
| 2 | 54 (15.6%) | 0.88 | 0.57 - 1.36 | 0.56 | 17 (13.1%) | 1.34 | 0.65 - 2.78 | 0.43 | 31 (14.0%) | 1.77 | 0.98 - 3.19 | 0.06 |
| 3 | 58 (16.8%) | 0.89 | 0.58 - 1.37 | 0.59 | 21 (16.2%) | 1.59 | 0.79 - 3.18 | 0.19 | 46 (20.8%) | 2.70 | 1.55 - 4.72 | 0.000* |
| 4 | 58 (16.8%) | 1.01 | 0.66 - 1.57 | 0.95 | 22 (16.9%) | 1.85 | 0.93 - 3.69 | 0.08 | 39 (17.7%) | 2.44 | 1.38 - 4.32 | 0.000* |
| 5 or more | 49 (14.1%) | 0.66 | 0.43 - 1.03 | 0.07 | 41 (31.5%) | 3.37 | 1.80 - 6.34 | 0.000* | 48 (21.7%) | 2.72 | 1.57 - 4.72 | 0.000* |
| **Interval between primary and advanced diagnosis** |  |  |  |  |  |  |  |  |  |  |  |  |
| Advanced at primary diagnosis | 89 (25.7%) | Ref. |  |  | 34 (26.2%) | Ref. |  |  | 54 (24.4%) | Ref. |  |  |
| =< 36 months | 134 (38.7%) | 1.11 | 0.80 - 1.53 | 0.53 | 48 (39.9%) | 1.01 | 0.63 - 1.61 | 0.97 | 70 (31.7%) | 0.91 | 0.62 - 1.34 | 0.63 |
| > 36 months | 123 (35.6%) | 1.17 | 0.84 - 1.62 | 0.36 | 48 (39.9%) | 1.16 | 0.72 - 1.85 | 0.54 | 97 (43.9%) | 1.60 | 1.10 - 2.33 | 0.01* |
| * *P*-value statistically significant  † Only BRAF-positive patients included |  |  |  |  |  |  |  |  |  |  |  |  |

| Table 3: Patient and tumor characteristics associated with systemic therapy use (multivariable logistic regression model, inclusion in the model only if statistically significant in univariable model) | | | | | | | | | | | | |
| --- | --- | --- | --- | --- | --- | --- | --- | --- | --- | --- | --- | --- |
|  | Immune therapy (N=346) | | |  | Targeted therapy† (N=130) | | |  | Both therapies† (N=221) | | |  |
|  | N (%) | Odds Ratio | 95% CI | P-value | N (%) | Odds Ratio | 95% CI | P-value | N (%) | Odds Ratio | 95% CI | P-value |
| **Age group** |  |  |  |  |  |  |  |  |  |  |  |  |
| ≤ 40 | 5 (1.5%) | 0.62 | 0.18 - 2.13 | 0.44 | 7 (5.4%) | 1.91 | 0.72 - 5.05 | 0.19 | 19 (8.6%) | 6.30 | 2.77 - 14.4 | 0.000* |
| 41-54 | 38 (11.0%) | 1.51 | 0.82 - 2.76 | 0.18 | 12 (9.2%) | 0.84 | 0.40 - 1.75 | 0.65 | 54 (24.4%) | 6.04 | 3.40 - 10.7 | 0.000* |
| 55-64 | 59 (17.0%) | 1.30 | 0.78 - 2.18 | 0.31 | 27 (20.8%) | 1.54 | 0.86 - 2.75 | 0.15 | 48 (21.7%) | 2.78 | 1.60 - 4.81 | 0.000* |
| 65-74 | 121 (35.0%) | 1.79 | 1.18 - 2.70 | 0.01* | 48 (36.9%) | 1.53 | 0.93 - 2.50 | 0.09 | 72 (32.6%) | 2.72 | 1.66 - 4.46 | 0.000* |
| ≥ 75 | 123 (35.5%) | Ref. |  |  | 36 (27.7%) | Ref. |  |  | 28 (12.7%) | Ref. |  |  |
| **Sex** |  |  |  |  |  |  |  |  |  |  |  |  |
| Male | 236 (68.2%) | Ref. |  |  | 70 (53.9%) | Ref. |  |  | 150 (67.9%) |  |  |  |
| Female | 110 (31.8%) | 0.57 | 0.40 - 0.82 | 0.002* | 60 (46.1%) | 1.56 | 1.05 - 2.30 | 0.03* | 71 (32.1%) |  |  |  |
| **Topography** |  |  |  |  |  |  |  |  |  |  |  |  |
| Skin | 306 (88.4%) | Ref. |  |  | 114 (87.7%) |  |  |  | 188 (85.1%) |  |  |  |
| Unknown primary site | 40 (11.6%) | 0.70 | 0.36 - 1.36 | 0.29 | 16 (12.3%) |  |  |  | 33 (14.9%) |  |  |  |
| **Morphology** |  |  |  |  |  |  |  |  |  |  |  |  |
| Superficial Spreading Melanoma | 153 (44.2%) | Ref. |  |  | 73 (56.2%) | Ref. |  |  | 121 (54.8%) | Ref. |  |  |
| Nodular Melanoma | 83 (24.0%) | 0.78 | 0.51 - 1.19 | 0.25 | 30 (23.1%) | 0.95 | 0.59 - 1.54 | 0.84 | 37 (16.7%) | 0.64 | 0.40 – 0.99 | 0.05* |
| Malignant Melanoma NOS | 76 (22.0%) | 0.86 | 0.48 - 1.53 | 0.61 | 24 (18.4%) | 0.54 | 0.32 - 0.89 | 0.02* | 59 (26.7%) | 0.95 | 0.58 - 1.53 | 0.83 |
| Other | 34 (9.8%) | 1.16 | 0.59 - 2.26 | 0.66 | 3 (2.3%) | 0.31 | 0.09 - 1.05 | 0.06 | 4 (1.8%) | 0.21 | 0.07 - 0.63 | 0.01* |
| **Synchronous versus metachronous** |  |  |  |  |  |  |  |  |  |  |  |  |
| Metachronous | 262 (75.7%) |  |  |  | 98 (75.4%) |  |  |  | 170 (76.9%) |  |  |  |
| Synchronous | 73 (21.1%) |  |  |  | 30 (23.1%) |  |  |  | 44 (19.9%) |  |  |  |
| Unknown | 11 (3.2%) |  |  |  | 2 (1.5%) |  |  |  | 7 (3.2%) |  |  |  |
| **Elevated serum LDH level (>250 U/L)** |  |  |  |  |  |  |  |  |  |  |  |  |
| Yes | 151 (43.6%) | 0.64 | 0.43 - 0.94 | 0.02* | 90 (69.2%) | 1.37 | 0.87 - 2.15 | 0.18 | 112 (50.7%) | 0.75 | 0.52 - 1.10 | 0.15 |
| No | 152 (43.9%) | Ref. |  |  | 36 (27.7%) | Ref. |  |  | 104 (47.1%) | Ref. |  |  |
| Unknown | 43 (12.5%) | 1.43 | 0.75 - 2.73 | 0.27 | 4 (3.1%) | 0.26 | 0.09 - 0.92 | 0.01* | 5 (2.2%) | 0.28 | 0.10 - 0.78 | 0.02* |
| **BRAF mutation** |  |  |  |  |  |  |  |  |  |  |  |  |
| Negative | 268 (77.5%) | Ref. |  |  | 0 (0.0%) | † |  |  | 0 (0.0%) | † |  |  |
| Positive | 32 (9.2%) | 0.03 | 0.02 - 0.04 | 0.000* | 130 (100%) |  |  |  | 221 (100%) |  |  |  |
| Unknown/Not tested | 46 (13.3%) | 0.25 | 0.15 - 0.41 | 0.000* | 0 (0.0%) |  |  |  | 0 (0.0%) |  |  |  |
| **ECOG performance status** |  |  |  |  |  |  |  |  |  |  |  |  |
| 0 | 126 (36.4%) | Ref. |  |  | 26 (20.0%) | Ref. |  |  | 110 (49.8%) | Ref. |  |  |
| 1 | 129 (37.3%) | 0.97 | 0.62 - 1.51 | 0.88 | 40 (30.8%) | 1.57 | 0.91 - 2.71 | 0.55 | 75 (33.9%) | 0.57 | 0.39 - 0.84 | 0.01* |
| 2 | 31 (9.0%) | 0.40 | 0.22 - 0.74 | 0.004* | 29 (22.3%) | 2.89 | 1.56 - 5.35 | 0.001* | 22 (9.9%) | 0.42 | 0.24 - 0.74 | 0.003* |
| 3 or above | 10 (2.9%) | 0.11 | 0.05 - 0.24 | 0.000* | 16 (12.3%) | 2.31 | 1.12 - 4.77 | 0.02* | 3 (1.4%) | 0.07 | 0.02 - 0.22 | 0.000* |
| Unknown | 50 (14.4%) | 0.20 | 0.11 - 0.35 | 0.000* | 19 (14.6%) | 1.23 | 0.62 - 2.44 | 0.55 | 11 (5.0%) | 0.15 | 0.07 - 0.30 | 0.000* |
| **Charlson comorbidity index** |  |  |  |  |  |  |  |  |  |  |  |  |
| 0 | 146 (42.2%) | Ref. |  |  | 59 (45.4%) |  |  |  | 123 (55.6%) | Ref. |  |  |
| 1 | 90 (26.0%) | 1.05 | 0.67 - 1.65 | 0.82 | 23 (17.7%) |  |  |  | 45 (20.4%) | 0.95 | 0.61 - 1.47 | 0.81 |
| 2 or more | 40 (11.6%) | 0.41 | 0.24 - 0.71 | 0.001* | 26 (20.0%) |  |  |  | 15 (6.8%) | 0.54 | 0.29 – 0.99 | 0.05* |
| Unknown | 70 (20.2%) | 0.94 | 0.58 - 1.54 | 0.81 | 22 (16.9%) |  |  |  | 38 (17.2%) | 0.97 | 0.60 - 1.56 | 0.89 |

| *Appendix III (continued)* |  |  |  |  |  |  |  |  |  |  |  |  |
| --- | --- | --- | --- | --- | --- | --- | --- | --- | --- | --- | --- | --- |
| **Socioeconomic status (SES)** |  |  |  |  |  |  |  |  |  |  |  |  |
| Low | 48 (13.9%) |  |  |  | 18 (13.9%) |  |  |  | 33 (14.9%) |  |  |  |
| Low-middle | 41 (11.8%) |  |  |  | 18 (13.9%) |  |  |  | 40 (18.1%) |  |  |  |
| Middle | 47 (13.6%) |  |  |  | 18 (13.9%) |  |  |  | 38 (17.2%) |  |  |  |
| Middle-high | 40 (11.6%) |  |  |  | 17 (13.0%) |  |  |  | 38 (17.2%) |  |  |  |
| High | 49 (14.1%) |  |  |  | 11 (8.4%) |  |  |  | 28 (12.7%) |  |  |  |
| Unknown | 121 (35.0%) |  |  |  | 48 (36.9%) |  |  |  | 44 (19.9%) |  |  |  |
| **Number of metastases** |  |  |  |  |  |  |  |  |  |  |  |  |
| None/Unknown | 68 (19.6%) |  |  |  | 15 (11.5%) | Ref. |  |  | 22 (10.0%) | Ref. |  |  |
| 1 | 59 (17.1%) |  |  |  | 14 (10.8%) | 0.72 | 0.33 - 1.58 | 0.41 | 35 (15.8%) | 0.89 | 0.46 - 1.73 | 0.73 |
| 2 | 54 (15.6%) |  |  |  | 17 (13.1%) | 0.96 | 0.45 - 2.08 | 0.93 | 31 (14.0%) | 0.90 | 0.45 - 1.79 | 0.77 |
| 3 | 58 (16.8%) |  |  |  | 21 (16.2%) | 1.00 | 0.47 - 2.11 | 0.99 | 46 (20.8%) | 1.39 | 0.72 - 2.67 | 0.33 |
| 4 | 58 (16.8%) |  |  |  | 22 (16.9%) | 0.94 | 0.44 - 1.99 | 0.86 | 39 (17.7%) | 1.15 | 0.58 - 2.27 | 0.70 |
| 5 or more | 49 (14.1%) |  |  |  | 41 (31.5%) | 1.60 | 0.78 - 3.26 | 0.20 | 48 (21.7%) | 1.22 | 0.63 - 2.40 | 0.55 |
| **Interval between primary and advanced diagnosis** |  |  |  |  |  |  |  |  |  |  |  |  |
| Advanced at primary diagnosis | 89 (25.7%) |  |  |  | 34 (26.2%) |  |  |  | 54 (24.4%) | Ref. |  |  |
| =< 36 months | 134 (38.7%) |  |  |  | 48 (39.9%) |  |  |  | 70 (31.7%) | 0.92 | 0.55 - 1.52 | 0.74 |
| > 36 months | 123 (35.6%) |  |  |  | 48 (39.9%) |  |  |  | 97 (43.9%) | 1.23 | 0.74 - 2.05 | 0.42 |
| * *P*-value statistically significant  † Only BRAF-positive patients included |  |  |  |  |  |  |  |  |  |  |  |  |
|  |  |  |  |  |  |  |  |  |  |  |  |  |
|  |  |  |  |  |  |  |  |  |  |  |  |  |
